# Design, delivery, and evaluation of early interventions for children exposed to acute trauma

**DOI:** 10.3402/ejpt.v5.22757

**Published:** 2014-07-02

**Authors:** Nancy Kassam-Adams

**Affiliations:** 1 Center for Injury Research and Prevention, Children's Hospital of Philadelphia, Philadelphia, PA, USA; 2 Center for Pediatric Traumatic Stress, Children's Hospital of Philadelphia, Philadelphia, PA, USA; 3 Department of Pediatrics, Perelman School of Medicine, University of Pennsylvania, Philadelphia, PA, USA

**Keywords:** prevention, early intervention, acute traumatic stress, children, adolescents

## Abstract

**Background:**

Exposure to acute, potentially traumatic events is an unfortunately common experience for children and adolescents. Posttraumatic stress (PTS) responses following acute trauma can have an ongoing impact on child development and well-being. Early intervention to prevent or reduce PTS responses holds promise but requires careful development and empirical evaluation.

**Objectives:**

The aims of this review paper are to present a framework for thinking about the design, delivery, and evaluation of early interventions for children who have been exposed to acute trauma; highlight targets for early intervention; and describe next steps for research and practice.

**Results and conclusions:**

Proposed early intervention methods must (1) have a firm theoretical grounding that guides the design of intervention components; (2) be practical for delivery in peri-trauma or early post-trauma contexts, which may require creative models that go outside of traditional means of providing services to children; and (3) be ready for evaluation of both outcomes and mechanisms of action. This paper describes three potential targets for early intervention—maladaptive trauma-related appraisals, excessive early avoidance, and social/interpersonal processes—for which there is theory and evidence suggesting an etiological role in the development or persistence of PTS symptoms in children.

Exposure to acute, potentially traumatic events is an unfortunately common experience for children and adolescents (Costello, Erkanli, Fairbank, & Angold, 2002). Millions of children each year experience violence, road traffic accidents, unintentional injury, fire, natural or manmade disasters, and terrorism. Although many children are resilient and recover well, posttraumatic stress (PTS) responses following acute trauma occur in a significant minority of these children, and, if persistent, these symptoms can have ongoing impact on child development and well-being (Copeland, Keeler, Angold, & Costello, 2007). Early intervention to prevent or reduce PTS responses holds promise, but the empirical literature for early interventions post-trauma has demonstrated that well-intentioned interventions can be ineffective in preventing psychological sequelae (Roberts, Kitchiner, Kenardy, & Bisson, 2009; Rose, Bisson, Churchill, & Wessely, 2001). This highlights the need for careful development and evaluation of early interventions for children.

For the purposes of this paper, “early intervention” refers to efforts undertaken in the peri-trauma and early post-trauma period to prevent or reduce the development, persistence, and severity of traumatic stress responses and to promote children's resilience and full emotional recovery after exposure to an acute, potentially traumatic event. This encompasses secondary prevention of negative sequelae given a trauma exposure, as well as possible early treatment of severe or impairing acute PTS symptoms. (Early intervention to treat acute PTS serves a dual purpose, in that reducing these early symptoms is likely to help prevent longer-term psychological sequelae.) Although PTS responses are the focus of this paper, some early interventions may also aim to prevent or reduce other specific types of psychological sequelae (e.g., depression symptoms). This paper's high-level summary will not detail specific developmental and cultural considerations in the design and implementation of early interventions for trauma-exposed children; however, both development and culture are extremely important. Intervention targets and methods must be culturally relevant, and those delivering them must be culturally competent. Developmental differences in cognitive, emotional, and social domains across young children, school-age children, and adolescents will affect early intervention targets and delivery choices, as well as the appropriate role of parents and caregivers in intervention activities.

Within the past decade, the empirical literature regarding early interventions for trauma-exposed children has begun to grow. There are now a small number of published, well-designed evaluation studies of early interventions for children that aim to prevent or reduce later traumatic stress responses. Several more randomized trials are underway at the time of this writing (Kenardy, Cobham, Nixon, McDermott, & March, 2010; Landolt, 2010; Marsac et al., 2013). Table 1 summarizes published trials of early interventions, as well as on-going trials which have published study protocols. Several things are notable. Nearly all of the existing studies have focused on school-age children and adolescents. The timing of interventions varies from peri-trauma (within hours of an acute event) to a month or more post-trauma. The vast majority included children exposed to injury or a road traffic accident; randomized or controlled trials of early interventions for children post-disaster or other mass trauma are nearly non-existent. The health care setting provides direct access to recently trauma-exposed children and the culture of this setting is conducive to performing screening, triage, and brief interventions. Most notably, few studies have found clear evidence of main effects on PTS symptoms. A recent meta-analysis of early intervention trials concluded that there is preliminary evidence that early intervention can be helpful but also found that the heterogeneity of studies made it difficult to draw clear conclusions about effective elements (Kramer & Landolt, 2011).

**Table 1:** Summary of early interventions for children exposed to acute traumatic events and randomized trial results

	Level of preventive care	Nature of intervention	Who delivers/Where delivered	When post-trauma	Type of event(s)	Child age	N in trial	Main effect on child PTSD	Other findings
Initiated in peri-trauma period									
‘So you've been in an accident’ booklet (Kenardy, Thompson, Le Brocque, & Olsson, 2008)	Universal	Informational booklet (parent–child)	Nurse/at hospital	72 h	Injury	7–15	103	no	Reduced anxiety symptoms
Stepped preventive care (SPC) (Kassam-Adams et al., 2011)	Stepped care model: Targeted/	One session plus phone follow-up, with option for more intensive services as needed. (parent–child)	Nurse or social worker/at hospital	Screen and initiate intervention within 1 week	Injury	8–17	85	no	Effective risk screening protocol
Preventive medication (Nugent et al., 2010)	Targeted	10-day trial of propranolol (child)	Physician/at hospital	Initiated within 12 h of admission	Injury	10–18	29	no	In treatment-adherent group: trend for reduced PTS in boys, increased PTS in girls
Preventive medication (Stoddard et al., 2011)	Universal	24-week course of sertraline (child)	Physician/at hospital	Initiated during hospitalization	Burn injury/post-burn reconstructive surgery	6–20	26	mixed	Reduced parent reported child PTS; No difference in self-reported child PTS
Initiated in early post-trauma period									
Individual psychological debriefing (Stallard et al., 2006)	Universal	One session (child)	Mental health professional/at hospital	Within 4 weeks	Road traffic accident	7–18	158	no	–
Classroom-based group intervention (Karam et al., 2008)	Universal	12 sessions on consecutive school days	Teacher/in classroom	1 month	War	6–18	194	no^a^	–
Kids and Accidents website (Cox & Kenardy, 2010)	Universal	Printed informational materials (parent–child) website (child)	Research staff/by mail plus website	Within 2 weeks	Road traffic accident	7–16		no	Reduced anxiety symptoms
Psychological interventions in children after road traffic accidents (PICARTA) (Zehnder, Meuli, & Landolt, 2010)	Universal	One session (parent–child)	Mental health professional/at hospital	10 days	Road traffic accident	7–16	99	no	Pre-teens: Reduced depression, behavior problems
Child and Family Traumatic Stress Intervention (CFTSI) (Berkowitz et al., 2011)	Targeted	Four sessions (parent–child)	Mental health professional	Screen within 1 month	Road traffic accident, assault, sexual abuse, violence, injury	7–17	106	yes	–
Child- and family-focused Cognitive-behavioral Early Intervention for PTSD (Kenardy et al., 2010)	Stepped care model: Indicated	Two-stage screening followed by child-focused (6 sessions) or family-focused (10 sessions) CBT if symptomatic at second screen	Mental health professional	Screen at 1–2 weeks and at 4–6 weeks; Treatment initiated after second screen	Injury	7–16	(140)	Trial is ongoing	–
Psychological interventions in children after road traffic accidents or burns (PICARTA-B) (Landolt, 2010)	Targeted	Two sessions (parent–child)	Mental health professional	Screen within 1 week; Intervention within 2 weeks	Road traffic accident, burn injury	2–16	(120)	Trial is ongoing	–
Coping Coach (Marsac et al., 2013)	Universal	Web-based intervention/game in three modules (child)	Introduced by research staff at hospital/Delivered online	Within 2 weeks	Acute medical event	8–12	(70)	Trial is ongoing	–

a Compared to children in schools not receiving the intervention. Schools could not be randomly assigned due to local authority mandating which schools received intervention.

Recent comprehensive reviews of interventions for children exposed to disaster (La Greca & Silverman, 2009) or armed conflict (Peltonen & Punamaki, 2010), and of school-based interventions for PTS symptoms (Rolfsnes & Idsoe, 2011) reveal almost no rigorous studies of interventions delivered in the early post-trauma period to prevent the development of psychological sequelae. One notable exception is a school-based intervention initiated 1 month post-war in Lebanon (Karam et al., 2008). This carefully designed controlled trial of an early intervention demonstrates some of the challenges of rigorous evaluation in post-trauma settings, in that random assignment to treatment condition was precluded by the mandate of local authorities that certain schools receive the intervention.

This nascent empirical literature provides a basis from which to move the field forward. The aims of this paper are to: (1) present a framework for thinking about the design, delivery, and evaluation of early interventions for children who have been exposed to acute trauma; (2) highlight three likely targets for early intervention; and (3) describe next steps for research and practice.

## Framework for thinking about early interventions for trauma-exposed children

How can we, as a field, work systematically to improve the effectiveness and reach of our overall “toolkit” of early interventions to prevent or reduce the severity of PTS in children following acute traumatic events? In this paper, I argue that in order to move the field forward, each proposed early intervention method must have three very important characteristics. First, it must have a firm theoretical grounding that guides the design or selection of intervention components. Second, it must be practical for delivery in peri-trauma or early post-trauma contexts, which may require creative models that go outside of traditional means of providing services to children. And third, it must be ready for evaluation to assess both outcomes and mechanisms of action.

### Design

There have been a number of calls to reclaim the central role of theory to guide trauma research (Benight, 2012), and intervention development (Feldner, Monson, & Friedman, 2007; Ruzek, 2008). For early intervention design, this means combining an explicit model of etiological processes in PTS symptom development with thoughtful selection of intervention methods that are likely to have impact in changing those specific processes (Feldner et al., 2007). Etiological models of child PTS development across the peri-trauma and early post-trauma period include (but are not limited to) social cognitive theory, information-processing theories, models of emotional regulation and coping, and models of the interplay of neurobiological processes with emotions and coping (Benight, 2012; Dalgleish, Meiser-Stedman, & Smith, 2005; Olff, Langeland, & Gersons, 2005; Skinner & Zimmer-Gembeck, 2007). Early interventions for acute child trauma can draw upon a growing evidence base regarding specific etiological factors that may be malleable in the peri- and post-trauma periods. In an iterative fashion, well-designed and carefully evaluated early interventions can play a crucial role in advancing our understanding of underlying etiological processes.

Regardless of the underlying theoretical model, there are several cross-cutting issues to be considered in the design of any early intervention for trauma-exposed children. One key issue is the timing of intervention. Appropriate targets and methods will vary in the peri-trauma period—during and immediately after the event—compared to the early post-trauma period in the first few weeks post-event. For example, in the peri-trauma period it may be possible and desirable to target aspects of the child's objective and subjective experience of the event itself (Kazak, Kassam-Adams, Schneider, Zelikovsky, & Alderfer, 2006). Another cross-cutting issue is the determination of whether an intervention is to be delivered as a universal preventive measure, as targeted prevention for children with some specified risk factor or characteristic, or as early indicated clinical treatment of specific symptoms (Feldner et al., 2007; Kazak et al., 2006). Stepped care models, which systematically combine these levels of intervention, have also shown promise.

### Delivery

Implementation of early intervention requires a thorough understanding of the trajectory of traumatic stress responses as well as careful consideration of the practical realities of reaching children in the early aftermath of an acute event. Careful consideration of where, how, and by whom early intervention can be delivered is integral to creating interventions that are both efficacious and wide-reaching. For example, depending on the target of intervention and the population one wishes to reach, an early intervention might optimally be delivered in person by a professional versus lay helper, online via a mobile or web-based application, or even via public health messaging that is broadly disseminated in traditional and social media.

Acute traumatic events that capture public attention are often those such as disasters or mass violence which affect whole communities or groups of children at once. These relatively less frequent events can affect large numbers of children or adolescents simultaneously, and involve varying degrees of disruption of community infrastructure that will limit the ability of children's normal support systems and service systems to provide assistance. Models such as Psychological first aid (PFA; National Child Traumatic Stress Network and National Center for PTSD, 2006) are explicitly designed to be implemented by helpers who are available (perhaps temporarily) in the early post-trauma period within this sort of context. On the contrary, many millions of children each year are exposed to acute events that affect one individual or family at a time (e.g., car crashes, residential fire, street violence, sudden medical events). These events occur with such frequency across the general population that the number of children exposed is quite high. Models such as the DEF Protocol for Pediatric Healthcare Providers (Kazak et al., 2006) are designed for implementation by helping professionals embedded in service systems (e.g., the health care system) that routinely see children during and immediately after acute traumatic events (i.e., in the peri-trauma period).

### Evaluation

All of the design and delivery considerations described here are also relevant to evaluation—a well-designed intervention is easier to evaluate, and any evaluation must take into account the specific timing, context, and mode of delivery of an intervention. To ensure that an early intervention method can be usefully evaluated, it is extremely helpful to describe it via a program theory or logic model which specifies intended target(s) and hypothesized mechanism(s) of action for each component of the intervention. Well-designed evaluations of early interventions for children, guided by an explicit program theory, can and should advance our understanding of underlying etiological processes in child traumatic stress. Even when randomized controlled trials (RCTs) are not feasible, careful study design can shed light on mechanisms of action.

Systematic implementation of early interventions for child acute trauma will require solid evidence about cost, reach, and effectiveness. Thus, for maximal public health and policy impact, wherever possible our evaluation studies should try to estimate the cost of interventions (in time, personnel, etc.) and evaluate population “reach” as well as clinical effectiveness (Zatzick, 2012).

## Targets for early intervention

Early interventions should target key etiological factors or processes involved in PTS symptom development or persistence in children that are malleable in the peri-trauma or early post-trauma period. The best evidence regarding these etiological processes would come from theoretically-grounded experimental studies or intervention studies that explicitly test mechanisms of action. In the absence of such studies, we can also learn from studies that identify predictors of child PTS outcomes after acute trauma, with the important caveat that predictors may not have a causal role in PTS development (Kraemer et al., 1997). In several recent meta-analyses, predictors with medium to large effect sizes included peri-trauma subjective life threat (Cox, Kenardy, & Hendrikz, 2008; Furr, Comer, Edmunds, & Kendall, 2010; Kahana, Feeny, Youngstrom, & Drotar, 2006); peri-trauma fear (Trickey, Siddaway, Meiser-Stedman, Serpell, & Field, 2012); early post-trauma psychological reactions (PTS, depression, or anxiety; Alisic, Jongmans, van Wesel, & Kleber, 2011; Furr et al., 2010; Kahana, et al., 2006; Trickey, et al., 2012); parents’ early PTS symptoms (Alisic et al., 2011; Cox et al., 2008); low post-trauma social support, that is, from parents, teachers, friends (Trickey et al., 2012); post-trauma poor family functioning (Trickey et al., 2012); and specific post-trauma coping strategies, that is, social withdrawal, distraction, thought suppression (Trickey et al., 2012).

This paper highlights three potential targets for early intervention—maladaptive trauma-related appraisals, excessive early avoidance, and social/interpersonal processes (notably social support and parent–child interactional processes). For each of these, we have theory and evidence suggesting an etiological role in the development or persistence of PTS symptoms in children, as well as evidence from intervention studies suggesting that these may be promising targets. This is not meant to be an exhaustive list, and other factors or processes may certainly be important as intervention targets. Additional studies of the etiology of PTS responses are needed to expand our understanding of potential targets, for example, acute emotional responses such as shame or anger.

Table 2 presents a number of early intervention models which have been proposed and described in the literature, and indicates whether each model directly addresses the three intervention targets highlighted here. As we progress as a field, it will be useful to systematically characterize the specific intervention target(s) addressed by proposed early intervention models and use the results of rigorous evaluation studies to determine which target(s) are most essential for preventing PTS.

**Table 2:** Proposed early intervention models/programs and selected intervention targets

	Intervention targets		
	
	Appraisals/interpretations^a^	Excessive avoidance^b^	Social/interpersonal^c^
Psychological first aid (PFA) (National Child Traumatic Stress Network and National Center for PTSD, 2006)	–	–	yes
DEF protocol for pediatric healthcare providers (Kazak et al., 2006)	–	–	yes
‘So you've been in an accident’ information booklet (Kenardy et al., 2008)	–	–	yes
Kids and Accidents website (Cox & Kenardy, 2010)	yes	–	yes
Stepped preventive care (SPC) (Kassam-Adams et al., 2011)	varies^d^	varies^d^	varies^d^
Child and Family Traumatic Stress Intervention (CFTSI) (Berkowitz, et al., 2011)	varies^d^	varies^d^	yes
Psychological interventions in children after road traffic accidents (PICARTA) (Zehnder et al., 2010)	yes	–	yes
Child- and family-focused cognitive–behavioral early intervention for PTSD (Kenardy et al., 2010)	yes	yes	varies^e^
Coping Coach web-based intervention (Marsac et al., 2013)	yes	yes	yes

a Intervention is designed to directly target maladaptive trauma-related appraisals or interpretations.

b Intervention is designed to directly target reduction of avoidance behaviors, thought suppression, or avoidance coping strategies.

c Intervention is designed to directly target social/interpersonal processes to increase social support or modify parent–child interactions.

d Intervention includes optional modules that may address this target for some children.

e One version of the intervention involves parents and targets family processes.

### Trauma-related appraisals

Early interventions might target trauma-related appraisals or interpretations, with the aim of reducing maladaptive appraisals, promoting adaptive appraisals, and/or enhancing a child's cognitive re-appraisal skills and related cognitive coping strategies.

Information-processing models suggest that maladaptive cognitive appraisals after a difficult event lead to behavioral strategies (i.e., coping) that directly produce traumatic stress symptoms and/or prevent the development of realistic and adaptive appraisals (Ehlers & Clark, 2000; Meiser-Stedman, 2002). As predicted by these models, children's maladaptive appraisals of a potentially traumatic medical event and of their own emotional reactions to the event do appear to be associated concurrently with acute PTS symptoms (Salmon, Sinclair, & Bryant, 2007), and predict the persistence of PTS symptoms (Ehlers, Mayou, & Bryant, 2003; Meiser-Stedman, Dalgleish, Glucksman, Yule, & Smith, 2009). Conversely, adaptive cognitive appraisals are associated with better emotional recovery (Ellis, 2008). This initial evidence is consistent with an etiological role for appraisals, and suggests that trauma-related appraisals and interpretations are a key target for early intervention. Based in cognitive–behavioral treatment models, many existing early intervention methods have targeted appraisals to some extent (see Table 2). Early interventions can explicitly teach participants to recognize and modify problematic appraisals. Future early intervention models might also include non-explicit methods of modifying cognitive biases in interpretation (Lester, Field, & Muris, 2011a, 2011b).

### Excessive early avoidance

Early interventions might target excessive early avoidance, with the aim of reducing avoidance behaviors, thought suppression, or avoidance coping strategies.

Information-processing models also posit that fear conditioning can be central to the development and maintenance of traumatic stress responses. For example, after a frightening acute event, a child's natural ongoing exposure to reminders of the event promotes accurate (re)learning of the realistic threat involved with a trauma reminder and eventually diminishes emotional distress. On the contrary, a child who uses excessive avoidance (e.g., via distraction, thought suppression, avoidant behaviors) in the early post-trauma period may reduce his/her immediate distress but inadvertently divert a natural recovery process. Persistent PTS symptoms have been associated with children's use of avoidance coping, distraction or thought suppression (Stallard & Smith, 2007; Stallard, Velleman, Langsford, & Baldwin, 2001; Trickey et al., 2012; Zehnder, Prchal, Vollrath, & Landolt, 2006). Many existing early intervention methods target avoidance to some extent (see Table 2). Early interventions can address children's newly developing trauma-related feared situations by teaching about the short-term gains but long-term costs of avoidance, building skills for identifying trauma-related triggers, and encouraging approach behaviors in safe but feared situations.

### Social/interpersonal processes

Early interventions might target social and interpersonal processes, with the aim of increasing effective social support available to the child, modifying parent–child interactions, or reducing social withdrawal and enhancing support-seeking as an early coping strategy.

There is extensive literature documenting the essential role of social bonds and social support in the aftermath of disasters and other acute trauma (Charuvastra & Cloitre, 2008; Norris et al., 2002). In the peri-trauma and early post-trauma period, the child's family and social environment will vary in the availability and effectiveness of accurate and timely support. Children are active participants in this process, and vary in the extent to which they seek support during and after an acute event. The coping strategy of seeking social support has been linked to reduced PTS symptoms in children (Stallard et al., 2001; Trickey et al., 2012) and, conversely, coping via social withdrawal is associated with greater risk of posttraumatic stress disorder (PTSD) (Trickey et al., 2012). Early interventions can address the capacity of a child’s social environment to provide support, and the child’s capacity to seek and to effectively use social support.

Social learning models also suggest key interpersonal processes involved in the development and maintenance of anxiety symptoms in children. For example, studies have identified patterns of parent–child interaction that promote (or challenge) children's maladaptive appraisals and avoidant coping strategies (Cobham et al., 2012; Dadds, Barrett, Rapee, & Ryan, 1996). Evidence from a recent meta-analysis supports the active involvement of parents in early intervention approaches (Kramer & Landolt, 2011). Many existing early interventions for children do involve parents to some extent (see Table 2) but only a few explicitly target parent–child processes (Berkowitz, Stover, & Marans, 2011).

## Next steps: Agenda for research and practice

Our research agenda is clear but challenging. We must commit to doing rigorous yet practical evaluations of early interventions for children. We know that attractive and reasonable early intervention methods may be ineffective, thus evaluation is essential to guide our practice. A huge gap in the knowledge base is the lack of rigorous studies examining the effectiveness of early interventions for children exposed to disasters, war or terrorism, and other mass trauma (La Greca & Silverman, 2009; Peltonen & Punamaki, 2010). The following recommendations for research are distilled from the discussion above.

1. Integrate design, delivery, and evaluation considerations from the beginning. A well-designed intervention is easier to evaluate. Evaluation design should consider the timing, context, and mode of delivery of an intervention.
2. Strive to have every early intervention trial include a test of mechanisms of action that can help identify active ingredients in the intervention, and ideally elucidate underlying etiological mechanisms in child traumatic stress development.
3. Incorporate assessments within effectiveness trials that can help to estimate the cost–benefit and the reach of early interventions.
4. Think beyond the RCT to conduct evaluations in peri- and post-trauma contexts in which an RCT would not be feasible, for example, after disaster or mass violence.
5. Prepare for the next disaster or mass trauma with templates for basic research designs that can be implemented quickly.

The agenda for practice is similarly challenging. With regard to the available evidence to guide our practice, we have promising methods and best practices but no clear answers yet. At present, in most settings and circumstances, the best option is to use models of early intervention with children that are grounded in a sound theoretical basis and in the available research evidence. But we must not settle for this as a permanent state of affairs. Practitioners can help move the field forward in several ways.

1. Conduct (and publish) practice-based evaluations of early interventions with children as a way to add to the knowledge base about implementation challenges and successes.
2. Continue to think beyond mental health settings and professionals. Consider how to enlist a range of helpers suitable to the contexts in which trauma-exposed children can be found.
3. Learn from evolving practice in challenging post-trauma settings to develop novel methods of delivery (in person, online, in other modalities) grounded in solid principles, and collaborate with evaluators to test them.

A final recommendation for both research and practice is to promote collaboration and learning across settings. Given the myriad of practical barriers for research and practice, collaborative endeavors across practice settings, research sites, institutions, and nations are essential to improving early intervention for trauma-exposed children.
